# Type 1 diabetes, its complications, and non-ischemic cardiomyopathy: a mendelian randomization study of European ancestry

**DOI:** 10.1186/s12933-023-02117-7

**Published:** 2024-01-13

**Authors:** Yunyue Zhao, Enxi Quan, Tao Zeng, Zhuoshan Huang, Yanting Luo, Long Peng, Suhua Li, Jinlai Liu, Yutian Chong, Hong Cao

**Affiliations:** 1 Department of Cardiology, The Third Hospital of Sun Yat-sen University, Guangzhou, 510630 China; 2 Department of Clinical Pharmacy, Guangzhou First People’s Hospital, Guangzhou, 511457 China; 3 Department of Infectious Diseases, Guangdong Key Laboratory of Liver Disease, The Third Affiliated Hospital of Sun Yat-sen University, Guangzhou, 510630 China

**Keywords:** Type 1 diabetes, Type 1 diabetes with complication, Non-ischemic cardiomyopathy, Mendelian randomization, Mediation analysis

## Abstract

**Background:**

Type 1 diabetes (T1D) is a significant risk factor for a range of cardiovascular diseases. Nonetheless, the causal relationship between T1D and non-ischemic cardiomyopathy (NICM) remains to be elucidated. Furthermore, the mechanisms responsible for the progression from T1D to NICM have not been definitively characterized.

**Objective:**

The aim of this study was to conduct a Mendelian randomization (MR) study to investigate the causal effects of T1D and its complications on the development of NICM. Additionally, this study aimed to conduct a mediation analysis to identify potential mediators within this correlation.

**Methods:**

Genetic variants were used as instrumental variables for T1D. The summary data for T1D were obtained from two genome-wide association study datasets. The summary data for T1D with complications and NICM were obtained from the Finnish database. Two-sample MR, multivariable MR and mediation MR were conducted in this study.

**Results:**

The study revealed a causal association between T1D, T1D with complications, and NICM (with odds ratios of 1.02, 95% CI 1.01–1.04, *p* = 1.17e-04 and 1.03, 95% CI 1.01–1.05, *p* = 3.15e-3). Even after adjusting for confounding factors such as body mass index and hypertension, T1D remained statistically significant (with odds ratio of 1.02, 95% CI 1.01–1.04, *p* = 1.35e-4). Mediation analysis indicated that monokine induced by gamma interferon may play a mediating role in the pathogenesis of T1D-NICM (mediation effect indicated by odds ratio of 1.005, 95% CI 1.001–1.01, *p* = 4.9e-2).

**Conclusion:**

The study demonstrates a causal relationship between T1D, its complications, and NICM. Additionally, monokine induced by gamma interferon may act as a potential mediator in the pathogenesis of T1D-NICM.

**Supplementary Information:**

The online version contains supplementary material available at 10.1186/s12933-023-02117-7.

## Key points

**Question:** 1 Does type 1 diabetes(T1D) have an independent causal relationship with non-ischemic cardiomyopathy (NICM)?

2 Which inflammatory factors or diseases mediate the development of NICM in T1D?

**The following findings were identified in a Mendelian randomization study:**

**Primary findings:** 1 There is an independent causal relationship between T1D and NICM. Additionally, the causal relationship between T1D with complications and NICM is demonstrated. 2 Monokine induced by IFN-γ (MIG) mediates the progression from T1D to NICM.

**Secondary findings:** BMI, hypertension, glomerular diseases, and MIG are causally associated with NICM.

**Meaning:** All the findings are first-time discoveries in Mendelian randomization studies. The study confirms the causal relationship between T1D and NICM, while accounting for confounding factors. The MIG serves as a potential target for new preventive measure and therapy.

**Supplementary Information:**

The online version contains supplementary material available at 10.1186/s12933-023-02117-7.

## Introduction

Type 1 diabetes (T1D) is a chronic disease associated with poor cardiac outcomes and an increased risk of premature mortality [1–3]. It accounts for approximately 5–15% of diabetes cases in high-income countries and about 2% in low- and middle-income countries [4]. The prevalence of T1D is increasing worldwide, showing variations across different countries and areas, potentially influenced by environmental variables [5–8].

Cardiovascular diseases are the leading cause of mortality in individuals with T1D [9]. Previous cohort studies have also suggested that T1D can increase the risk of cardiovascular diseases [10–12]. For example, a recently published Mendelian randomized(MR) study indicated that T1D increases the risk of atherosclerosis [13]. Furthermore, Marcus Lind et al. showed that heart failure (HF) is a common complication in T1D patients [14]. However, there is an ongoing debate regarding the specific phenotype of HF associated with T1D. Most studies have suggested that T1D mainly affects the diastolic function, while effects on systolic function remain controversial [15–19]. Most cases in these studies were accompanied by confounding factors such as coronary artery disease and hypertension. According to the research on T1D conducted by Konduracka et al., it was found that the occurrence of HF and myocardial dysfunction was observed only in those who developed hypertension or coronary heart disease [20]. In a recent study, no significant differences in echocardiographic findings were observed between patients with T1D and healthy individuals, despite the presence of microvascular damage [21]. Therefore, the influence of T1D on HF, especially non-ischemic cardiomyopathy (NICM), remains incompletely understood based on human studies. Diabetic cardiomyopathy has been proposed as an explanation for the residual risk of HF in diabetic patients after accounting for coronary heart disease, hypertension and other factors [11]. However, most of the studies on T1D-induced diabetic cardiomyopathy have focused mainly on animal and cellular experiments [22–25]. Although diabetic cardiomyopathy is classified as a NICM resulting from diabetes mellitus, it is noteworthy that the myocardial pathologic phenotypes of T1D and type 2 diabetes (T2D) cardiomyopathy differ. Additionally, conducting real-world studies on T1D-induced NICM presents challenges in controlling for confounding factors. To address these gaps, it is essential to assess the causal relationship between T1D and NICM by MR method. In addition, another noteworthy consideration pertains to identify factors that mediate T1D-induced NICM. Previous observational studies have identified several inflammatory factors, such as interleukin-6, tumor necrosis factor α, and C-reactive protein (CRP), that are associated with HF [26, 27]. However, conflicting results have also been reported in some studies [28, 29]. Additionally, a observational study has shown that factors like renal disease and anemia are associated with the risk of HF [30]. Thus, we aim to investigate whether inflammatory cytokines and certain diseases have mediating roles in the development of T1D-induced NICM.

Conventional observational studies are susceptible to confounding factors and reverse causation bias. To overcome these limitations, MR utilizes genetic variants as instrumental variables (IVs) to infer causal relationships [31]. MR can not only overcome the limitations of observational studies by mimicking a randomized controlled trial but also provide evidence beyond clinical studies to establish the causal association between T1D and NICM. In this study, we performed two-sample MR analyses and multivariable MR (MVMR) to investigate the independent causal effect of T1D and its complications on NCIM. Furthermore, we conducted mediation analysis to explore the mediators in the association between T1D and NICM.

## Methods

### Two sample MR and MVMR

**Fig. 1:**
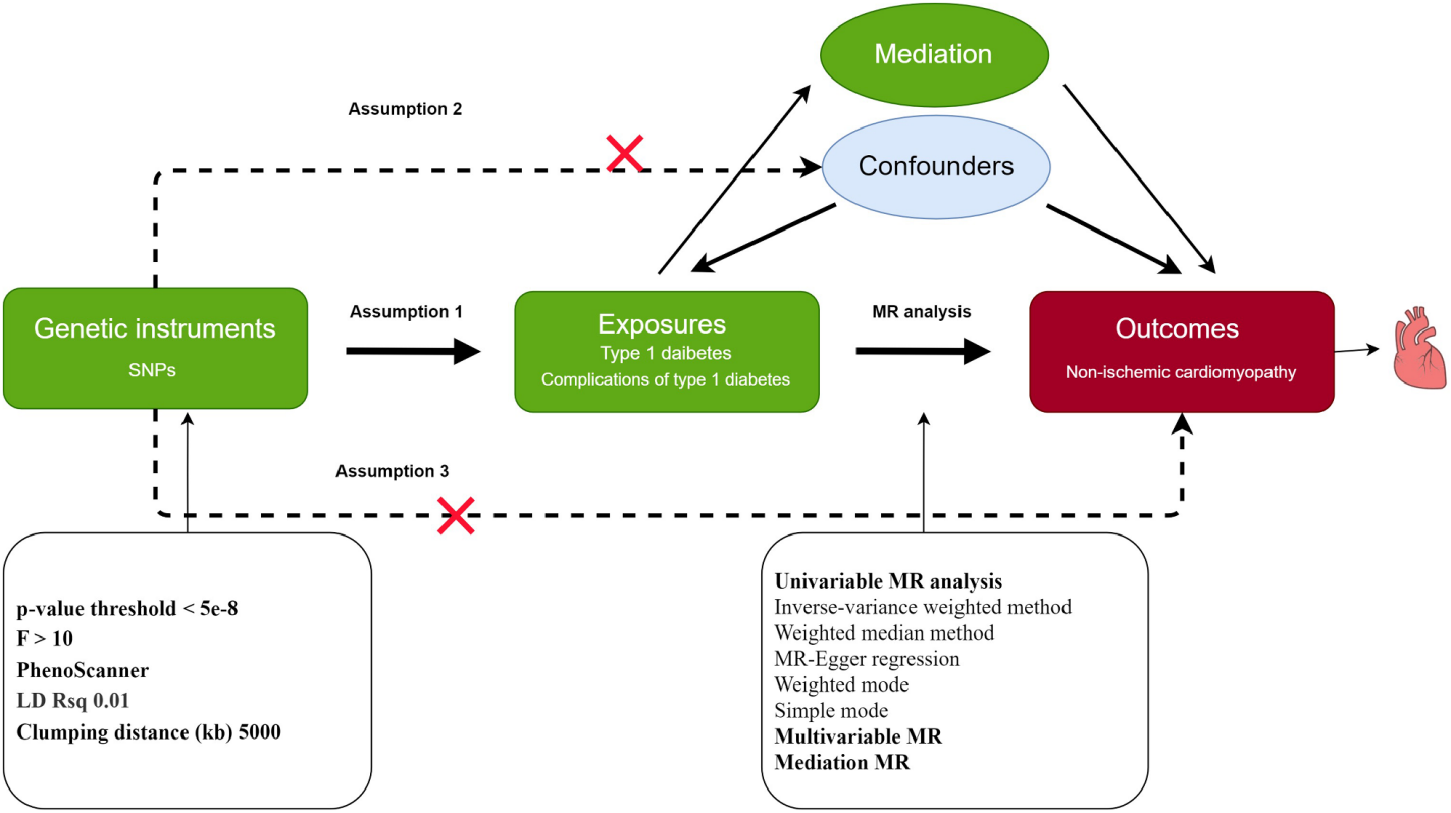
Study design

Figure 1 presents the study design. We used two-sample MR to investigate the causal effects of T1D and its complications on NICM [32]. To obtain the necessary data, we collected summary statistics from publicly available databases, as outlined in Table 1. Our single nucleotide polymorphisms (SNPs) selection process focused on SNPs strongly associated with T1D and randomly allocated at conception, ensuring minimal influence from environmental factors [33]. We followed three assumptions for MR analysis: (1) the selected IVs must be strongly associated with T1D; (2) the selected IVs should not be associated with potential confounders; (3) the selected IVs could only influence the NICM through T1D, but not other pathways. In the primary analysis, we conducted MR analysis using data from two T1D datasets and used the conventional random effect inverse variance weighted (IVW) method to estimate the causal effect of T1D on NICM. In addition, we also performed four complementary methods, including the weighted median method, the weighted mode method, simple mode, MR Egger. To ensure the robustness of the outcomes, we performed a meta-analysis of the results from two T1D datasets. We also conducted MVMR to mitigate potential pleiotropy by accounting for confounding factors such as body mass index (BMI) and hypertension. The analytic process adhered to the STROBE-MR guidelines [34].

**Table 1:** Information on data included in the study

Phenotypes/ID	Data source	Study information/PMID	Cases/controls	Author/Year
T1D: ebi-a-GCST010681	12 cohorts^#^	European /32,005,708	9266/15,574	Forgetta V/2020
T1D: ebi-a-GCST90018925	UKB, Finnish database	European/34,594,039	6447/451,248	NA/2022
T1D with complications: DM1NASCOMP	Finnish database	European	6234/308,280	NA/2022
T1D without complications: E4_DM1NOCOMP	Finnish database	European	4918/183,185	NA/2021
T1D with renal complications: E4_DM1REN	Finnish database	European	1579/308,280	NA/2022
T1D with ketoacidosis: E4_DM1KETO	Finnish database	European	2102/308,280	NA/2022
T1D with coma: E4_DM1COMA	Finnish database	European	2050/308,280	NA/2022
T1D with neurological complications: E4_DM1NEU	Finnish database	European	1077/308,280	NA/2022
T1D with peripheral circulatory complications: E4_DM1PERIPH	Finnish database	European	669/308,280	NA/2022
T1D with ophthalmic complications: E4_DM1OPTH	Finnish database	European	5202/308,280	NA/2022
Non-ischemic cardiomyopathy: finn-b-I9_NONISCHCARDMYOP	Finnish database	European	11,400/175,752	NA/2022
Hypertension: ukb-b-12,493	UKB	European	54,358/408,652	2018/Ben Elsworth
Body mass index: ukb-b-19,953	UKB	European	461,460	2018/Ben Elsworth
Monokine Induced by Gamma Interferon	YFS and FINRISK 1997 and 2002	European/33,491,305	8293	2020/Vanessa Tan

#, See Table S1 for more details. UKB (UK BioBank), YFS (Young Finns Study), FINRISK (Finland’s National FINRISK Study)

### Mediation MR/Two-step MR analysis

In the mediation analysis, we included glomerular disease, anemia, BMI, and hypertension. Furthermore, we included glycated hemoglobin, HOMA-IR, fasting insulin, blood lipids, CRP, and 41 other inflammatory factors in the mediation analysis. The three-step method provides evidence of a mediating role for a variable in the exposure-outcome effect. The indirect effect of each mediator was derived using the two-step MR method [35]. In the first step, we estimated the causal impact of T1D on a hypothesized mediator using IVs for T1D. In the second step, we established the causal impact of the mediators on NICM using IVs for the mediator. For all mediators individually, we quantified the proportion mediated by dividing the indirect effect by the total effect. Confidence intervals were estimated using the delta method [31].

### The data source and the selection of instrumental variables

We extracted summary-level data for the associations of SNPs with T1D from two Genome-Wide Association Studies (GWASs). One is a meta-analysis including 9,266 T1D cases and 15,574 non-cases from 12 European cohorts [36]. The other dataset is derived from the Finnish database and UKB data, consisting of 6,447 cases and 451,248 controls [37]. T1D with complications dataset obtained from Finnish database [38]. The NICM dataset comes from a Finnish database and contains 11,400 cases and 175,752 controls. For inflammatory cytokines, the data was from the study providing genome variant associations with 41 cytokines and growth factors in 8,293 individuals. This study combined the results from The Cardiovascular Risk in Young Finns Study (YFS) and FINRISK surveys [39]. The average participant ages are 37 years for YFS study and 60 years for FINRISK survey. Diseases in the Finnish database were diagnosed using ICD coding. The age distribution of patients and the inclusion process in the Finnish database can be accessed online through the link https://r9.risteys.finngen.fi/endpoints/+ID, such as ID E4_DM1PERIPH. Detailed information about the data sources can be found in Table 1 and Table S1. Table S1 includes information on all datasets and the available diagnostic codes.

We used strict selection criteria to select valid and reliable IVs for T1D. First, we searched for the largest GWAS summary statistics for the genetic proxies of T1D. We extracted SNPs strongly associated with T1D as candidate IVs (*p* < 5e-8). Second, we eliminated SNPs that were in linkage disequilibrium (r^2^ < 0.01) or palindromic with intermediate allele frequencies. Third, we excluded SNPs that were not available in the outcome GWAS or had proxy SNPs. In this study, we identified BMI and hypertension as confounding factors for NICM. We calculated the F statistics to measure the strength between IVs and T1D. We only considered SNPs with an F statistic > 10 as valid and reliable IVs for T1D. Finally, we included the 50 qualified SNPs as IVs to conduct the MR analysis. We extracted IVs of complications of T1D using the same method. Detailed information on those IVs is shown in Supplementary Excel 1. Since only few SNPs were identified for part of mediators when they were as the exposure, a higher cutoff (*p* < 5e-6) was chosen (*p* < 5e-6, Supplementary Excel 2).

### Statistical analysis

The MR estimates were represented by odds ratios (OR) with 95% confidence intervals (CIs). We performed the MR-Egger regression method, the leave-one-out method, and the MR-PRESSO method as sensitivity analysis. We used the MR-egger regression and MR-PRESSO method to test and correct the potential horizontal pleiotropy of the selected IVs. The MR-egger intercept and zero difference could indicate directional pleiotropy. The MR-PRESSO could detect and remove outliers in the IVs. We employed Cochrane’s Q statistic to evaluate the variability of SNPs estimates within each MR association. We used the p-value of the intercept test from MR-Egger regression to assess the horizontal pleiotropy [40]. By using MVMR analysis to adjust for confounding risk factors, we reduced the impact of confounding factors on the causal relationship. We performed all tests using the Two Sample MR [41], MR-PRESSO [42] and Mendelian Randomization [43] packages in the R software (version 4.0.2).

## Result

Univariable MR analysis supported a causal role for liability to T1D in the development of NICM. (IVW: GCST010681: OR 1.02; 95% CI 1.01–1.04; *p* = 1.17e-4; GCST90018925: OR 1.06; 95% CI 1.03–1.09; *p* = 0.02; Meta-analysis: OR 1.03; 95% CI 1.01–1.04; p<1e-4). Additionally, under sensitivity analyses, the other three methods, including MR-Egger, weighted median, and weighted mode, also revealed significant associations between T1D and NICM in GCST010681 and meta-analysis. Only the simple mode was attenuated (GCST010681: OR 1.02; 95% CI 0.99–1.06; *p* = 0.17; GCST90018925: OR 0.99; 95% CI 0.92–1.06; *p* = 0.68; Meta-analysis: OR 1.02; 95% CI 0.99–1.05; *p* = 0.29).

No heterogeneity or pleiotropy was observed in the associations between T1D (GCST010681) and NICM (*p* for heterogeneity = 0.35, *p* for pleiotropy = 0.28, respectively). For GCST90018925, heterogeneity exists but there is no evidence of pleiotropy (p for heterogeneity = 0.01, p for pleiotropy = 0.34, respectively). The results were robust in the leave-one-out and MR-PRESSO tests. To further rule out the influence of confounding factor level pleiotropy, we conducted MVMR. After matching for BMI, hypertension or both, statistical significance remained between T1D and NICM (Fig. 3). For additional information and visual representations of the data analysis, please refer to Supplementary Fig. 1, which includes scatter plots for the pleiotropy analysis, forest plots using the leave-one-out method, and funnel plots.

**Fig. 2:**
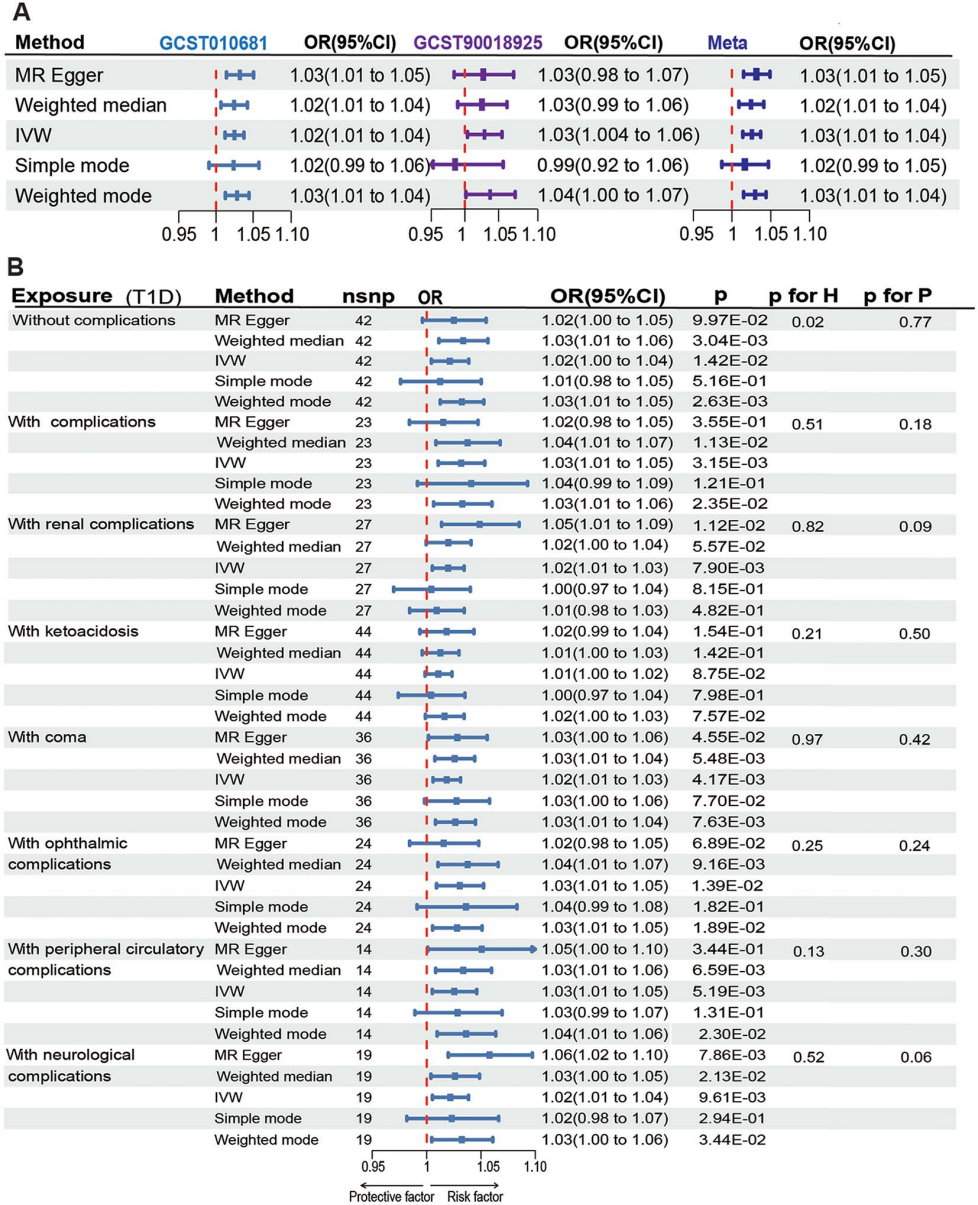
Genetically predicted type 1 diabetes and its complications: associations with the non-ischemic cardiomyopathy. IVW (Inverse Variance Weighted), H (Heterogeneity), P (Pleiotropy), CI (Confidence Interval), OR (Odds Ratio), *p* < 0.05 was considered statistically significant. The FDR-corrected results of the p-values (IVW) in each sub-group remained consistent with the uncorrected results

**Fig. 3:**
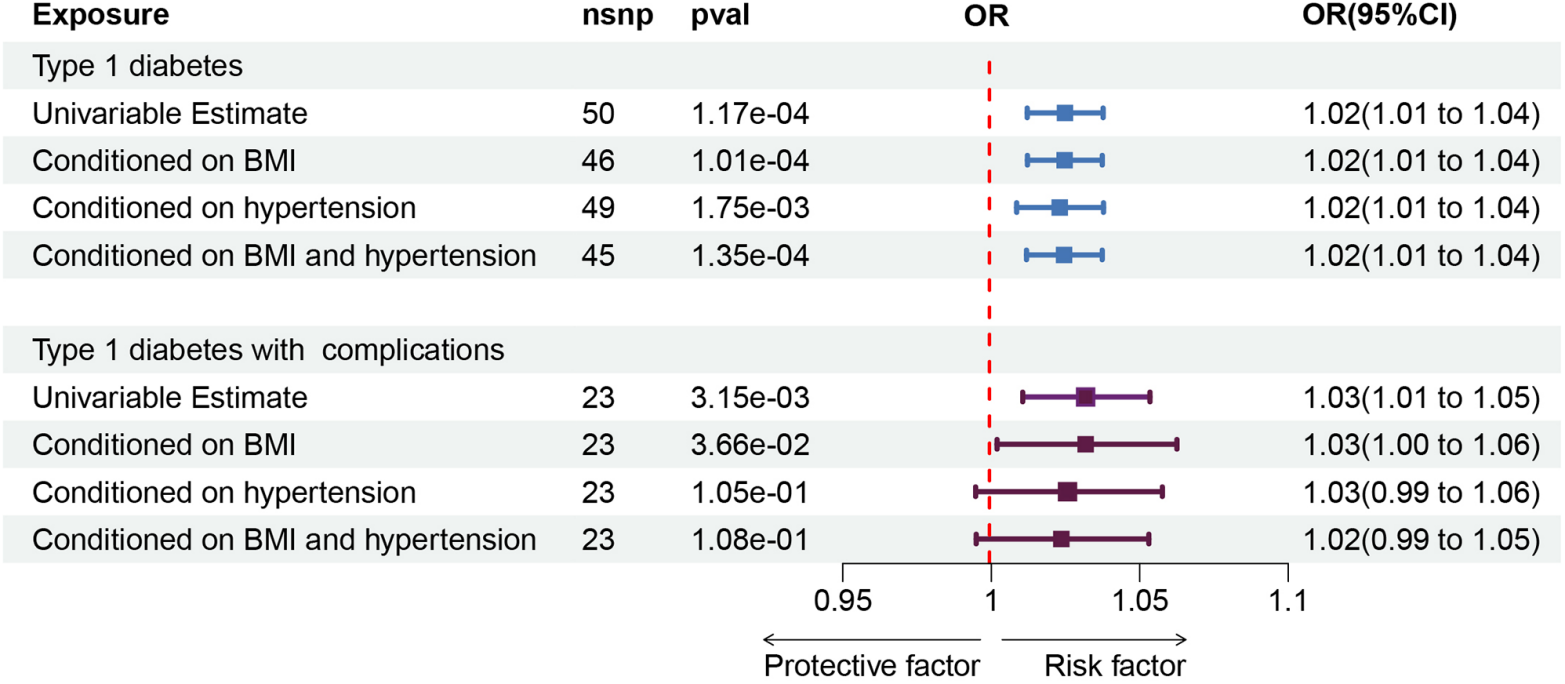
Genetically predicted association of T1D and its complications: associations with the non-ischemic cardiomyopathy after adjusting for confounders. CI (Confidence Interval), OR (Odds Ratio), *p* < 0.05 was considered statistically significant

To understand the relationship between different subgroups of T1D and NICM, we analyzed data from the Finnish database, which is the most comprehensive for T1D complications. Both T1D without complications and T1D with complications showed causal correlations with NICM (IVW: OR 1.02; 95% CI 1.004–1.04; *p* = 1.42e-02; OR 1.03; 95% CI 1.01–1.05; *p* = 3.15e-3, respectively). T1D with complications encompasses a range of diseases. These subgroup analyses also revealed significant causal correlations with NICM. The ORs of NICM were 1.02 (95% CI 1.01–1.03; *p* = 7.90e-03) for T1D with renal complications, 1.01 (95% CI 1.00-1.02; *p* = 8.75e-02) for T1D with ketoacidosis, 1.02 (95% CI 1.02–1.03; *p* = 4.17e-03) for T1D with coma, 1.03 (95% CI 1.01–1.05; *p* = 1.39e-02) for T1D with ophthalmic complications, 1.03 (95% CI 1.01–1.05; *p* = 5.19e-03) for T1D with peripheral circulatory complications, 1.02 (95% CI 1.01–1.04; *p* = 9.61e-03) for T1D with coma. Except for the analysis for T1D without complications, where heterogeneity was observed, all other subgroup analyses showed no significant heterogeneity or pleiotropy (Fig. 2). The results were robust in the leave-one-out and MR-PRESSO tests. We also conducted MVMR for T1D with complications. After matching for BMI, statistical significance remained (OR1.03, 95% CI 1.002–1.06, *p* = 3.66e-02) (Fig. 3). However, after adjusting for hypertension, the statistical correlation disappeared. In the subgroup analysis, the exposure and outcome datasets were from the same database. Therefore, there is a significant overlap in the control group. We used https://sb452.shinyapps.io/overlap to estimate the potential for Type I errors. After evaluation, even if the samples completely overlapped, the type I errors rate still be maintained at 0.05 in all subgroup analyses.

**Fig. 4:**
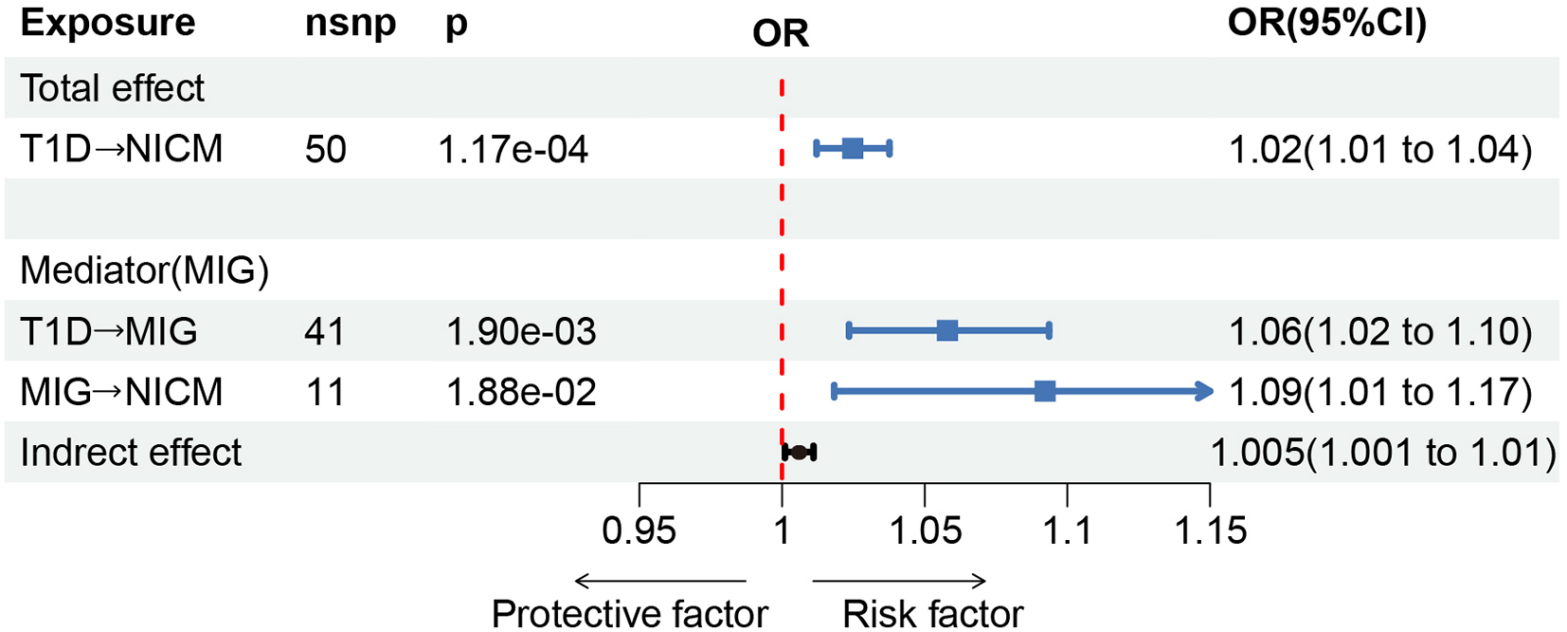
Mediation analysis. CI (Confidence Interval), OR (Odds Ratio), *p* < 0.05 was considered statistically significant. After applying FDR correction, the p-value for the correlation between MIG and NICM was determined to be 0.25

We then performed mediation analysis involving potential mediators, including anemia, glomerular disease, BMI, hypertension, glycated hemoglobin, HOMA-IR, fasting insulin, low-density lipoprotein cholesterol, triglyceride, intermediate-density lipoprotein and very-low-density lipoprotein. However, none of these factors demonstrated a mediating effect (Supplementary Excel.2). Among analyzed CRP and 41 inflammatory cytokines, a causal relationship with NICM was only found for Nerve Growth Factor and MIG. As Nerve Growth Factor had only 4 SNP instrumental variables, thus further analysis was not performed. Conversely, MIG mediated the relationship between T1D and NICM with an OR of 1.005 (95% CI 1.001–1.01) and accounted for 20% of the mediation effect (See Fig. 4). During the MR process, multiple tests were performed, hence the p-value was adjusted using false discovery rate (FDR) correction. The significance of the p-value for MIG disappears after correction (Supplementary Excel. 1).

## Discussion

The study provided genetic evidence supporting the causal association between NICM and T1D in univariable MR and MVMR analyses. Furthermore, the study demonstrated the causal relationship between T1D complications and NICM. Notably, there was no significant difference in the OR of NICM between T1D alone and T1D with complications.

Clinical observational studies have suggested an association between diabetes mellitus and HF [21, 44–46]. However, these studies primarily focus on T2D and are influenced by numerous confounding factors. For most diabetic patients who develop HF, their HF is related to coronary artery disease [18]. Therefore, it is necessary to elucidate the isolated impact of T1D on NICM. The impact of T1D on the myocardium is primarily focused on animal studies. In recent studies, it has been suggested that factors such as oxidative stress, inflammatory response, calcium ion imbalance, and energy dysregulation are involved in the impact of diabetes mellitus on the myocardium [47, 48]. Our previous study demonstrated impaired diastolic function in T1D SD rats [49]. Clinical research on the relationship between T1D and NICM is limited due to challenges in conducting prospective clinical studies, including cost and confounding biases. To address these challenges, we used an MR study to establish a causal connection between T1D and NICM, providing valuable evidence.

Anemia and nephropathy are relatively common concurrent diseases in patients with HF. Both of these conditions increase the risk factors for poor prognosis in patients with HF [50, 51]. Additionally, iron deficiency anemia and chronic kidney disease have been identified as risk factors for HF [30, 52]. However, it is worth noting that most research findings in the existing literature are derived from developed countries and largely focus on cases of ischemic cardiomyopathy. In a study from a developing country, the authors observed a significantly lower prevalence of anemia and nephropathy in individuals with NICM compared to studies conducted in Western countries [53]. An MR study suggested bidirectional causality between anemia and chronic HF [54]. Glomerular disease is a common complication of T1D. The correlation between T1D and anemia is unclear, but its complication, diabetic nephropathy, can cause anemia. The current study confirmed a causal association between genetically predicted T1D and genetically predicted glomerular disease as well as anemia. However, the causal relationship between anemia and NICM showed pleiotropy in the MR analysis. Although there is a causal relationship between glomerular disease and NICM, the mediating effect did not reach statistical significance. Therefore, further research is needed to analyze this potential mediating effect.

The association between inflammation and HF is currently a topic of great interest. An observational study conducted in 1990 found that patients with HF had elevated level of pro-inflammatory cytokines compared to healthy individuals [55]. Subsequent experimental and clinical research has highlighted the activation of the innate and adaptive immune systems as important factors in acute and chronic HF, leading to the exploration of potential immunotherapy for HF [56]. However, the outcomes of immunotherapy for HF have been less than satisfactory [57–59]. The CANTOS trial, a double-blind, randomized, placebo-controlled outcomes trial involving 10,061 patients with myocardial infarction and inflammatory atherosclerosis characterized by high-sensitivity CRP levels ≥ 2 mg/l, demonstrated a 15% reduction in the risk of the composite endpoint of non-fatal myocardial infarction, non-fatal stroke, or cardiovascular death compared to placebo [60]. Further exploration of this study revealed that patients with evidence of clonal hematopoiesis of indeterminate potential owing to mutations in TET2 had an improved response to canakinumab treatment compared with patients without the mutations [61]. This study provides inspiration that immunotherapy may not be universally effective for all cases of HF, and thus, it is important to explore which specific types of HF may respond positively to immunization. In an MR study, it was proposed that genetically predicted 10 inflammatory biomarkers (not including MIG) did not show a significant association with HF [28]. In current study, we investigated the causal relationship between 42 inflammatory biomarkers and discovered that MIG has a suggestive causal relationship with NICM and may plays a mediating role in the process of T1D causing NICM. Previous studies have also found that MIG is involved in immune checkpoint inhibitor myocarditis and chronic rejection after heart transplantation [62, 63]. Further exploration is warranted to determine the role of MIG in NICM.

### Strengths and limitations

To our knowledge, this is the first study to investigate the causal associations between T1D and NICM using univariable MR and MVMR analysis. The study fills a gap in the current human-level research on the causal relationship between T1D and NICM. Additionally, by investigating potential mediators, we can improve our understanding of the potential mechanisms underlying NICM, paving the way toward the development of preventative and therapeutic solutions. The application of the MR method helped to reduce confounding biases and derive robust causal effect estimates. Multiple sensitivity analyses and IV strength evaluations were conducted to ensure the reliability of the results. However, this study has certain limitations. Firstly, most of the data used in this study comes from individuals of European ancestry, which may limit the generalizability of our findings. Secondly, while subgroup MR analysis of T1D with complications can offer us a comprehensive insight into the association between various complications and NICM, it is important to acknowledge the considerable sample overlap between participants in the exposure and outcome datasets. In fact, these are single-sample analysis. This might increase the risk of type I errors, so caution should be exercised when interpreting the results in this section. We used https://sb452.shinyapps.io/overlap to estimate the potential for Type I errors. After evaluation, even if the samples completely overlapped, the type I error rate could be maintained at 0.05 in all subgroup analyses. Thirdly, even with the assurance of F statistic > 10, the explanatory power of IVs on potential mediating variables is limited. Therefore, even though many inflammatory cytokines have not been found to have a mediating effect, further research is still warranted in this area. In addition, the correlation p-value of MIG becomes non-significant after FDR correction. This suggests that it may play a mediating role, but more evidence is needed to confirm this.

## Conclusion

In conclusion, the study suggests that genetically predicted T1D and its complications play an independent causal role in the development of NICM. MIG may mediate the progression from T1D to NICM.

### Electronic supplementary material

Below is the link to the electronic supplementary material.

**Supplementary Material 1: Supplemental Excel 1**. Detailed data for univariate and multivariate mendelian randomization

**Supplementary Material 2: Table S1**. Descriptive information for all the datasets included and their corresponding diagnostic codes

**Supplementary Material 3: Supplemental Excel 2**. Detailed data for mediation mendelian randomization

**Supplementary Material 4:Supplemental Figure 1**. Visualization of Mendelian Randomization for T1D and T1D with Complications: A) Pleiotropy Analysis; B) Stability Analysis Utilizing the Leave-One-Out Method; C) Forest Plot Showing MR Effect Sizes Using MR-Egger and IVW; D) Funnel Plot

## Abbreviations

IVW: Inverse variance weighted
IVs: Instrumental variables
SNPs: Single Nucleotide Polymorphisms
FDR: False Discovery Rate
MVMR: Multivariable MR
T1D: Type 1 diabetes
T2D: Type 2 diabetic
NICM: Non-ischemic cardiomyopathy
HF: Heart failure
OR: Odds ratio
MIG: Monokine Induced by Gamma Interferon
HOMA-IR: Homeostatic Model Assessment of Insulin Resistance
GWAS: Genome-Wide Association Study
CRP: C-reactive protein
UKB: UK Biobank
