# Supplementary material for: Type 1 diabetes, its complications, and non-ischemic cardiomyopathy: a mendelian randomization study of European ancestry
Source: Cardiovasc Diabetol. 2024 Jan 13;23:31. doi: 10.1186/s12933-023-02117-7 (PMC10787423; doi:10.1186/s12933-023-02117-7)
Supplement: Supplementary file 2 — Supplementary Material 2: Table S1. Descriptive information for all the datasets included and their corresponding diagnostic codes [file 12933_2023_2117_MOESM2_ESM.docx]

Supplementary table 1: Information on data included in the study

| Phenotypes/ID | Data source | Study information/PMID | Cases/controls | Author/Year |
| --- | --- | --- | --- | --- |
| Type 1 diabetes:  ebi-a-GCST010681 | GoKinD,WTCCC^#^ | 12 European cohorts/32005708 | 9266/15574 | Forgetta V/2020 |
| Type 1 diabetes:  ebi-a-GCST90018925 | UKB, Finnish database | European/34594039 | 6447/451248 | NA/2022 |
| Type 1 diabetes with complications  finn-b-[E4_DM1NASCOMP](https://gwas.mrcieu.ac.uk/datasets/finn-b-E4_DM1NASCOMP/) | Finnish database | European | 6234/308280 | NA/2022 |
| Type 1 diabetes without complications  [finn-b-E4_DM1NOCOMP](https://gwas.mrcieu.ac.uk/datasets/finn-b-E4_DM1NOCOMP/) | Finnish database | European | 4918/183185 | NA/2021 |
| Type 1 diabetes with renal complications  finn-b-E4_DM1REN | Finnish database | European | 1579/308280 | NA/2022 |
| Type 1 diabetes with ketoacidosis  finn-b-E4_DM1KETO | Finnish database | European | 2102/308280 | NA/2022 |
| Type 1 diabetes with coma  finn-b-E4_DM1COMA | Finnish database | European | 2050/308280 | NA/2022 |
| Type 1 diabetes with neurological complications  E4_DM1NEU | Finnish database | European | 1077/308280 | NA/2022 |
| Type 1 diabetes with peripheral circulatory complications  E4_DM1PERIPH | Finnish database | European | 669/308280 | NA/2022 |
| Type 1 diabetes with ophthalmic complications  E4_DM1OPTH | Finnish database | European | 5202/308280 | NA/2022 |
| Non-ischemic cardiomyopathy: finn-b-I9_NONISCHCARDMYOP | Finnish database | European | 11400/175752 | NA/2022 |
| Hypertension:  ukb-b-12493 | UKB | European | 54358/408652 | 2018/Ben Elsworth |
| Body mass index  [ukb-b-19953](https://gwas.mrcieu.ac.uk/datasets/ukb-b-19953/) | UKB | European | 461460 | 2018/Ben Elsworth |
| Anaemias  finn-b-D3_ANAEMIA | Finnish database | European | 27371/88536 | NA/2022 |
| Glomerular disorders  finn-b-N14_GLOMEINOTH | Finnish database | European | 5642/371635 | NA/2022 |
| Cholesterol levels in IDL  ebi-a-GCST90092831 |  | European/35213538 | 115082 | Richardson TG/2022 |
| Glycated hemoglobin levels  ebi-a-GCST004939 |  | European/28887542 | 9436 | Prins BP/2017 |
| Fasting insulin  ebi-a-GCST90002237 |  | East Asian/34059833 | 29792 | Chen J/2021 |
| [Concentration of VLDL particles](https://gwas.mrcieu.ac.uk/datasets/ebi-a-GCST90093000/)  ebi-a-GCST90093000 |  | European/35213538 | 115,082 | Richardson TG/2022 |
| Triglyceride  ebi-a-GCST90014014 | UKB | European/34017140 | 389562 | Mbatchou J/2021 |
| LDL cholesterol  [ieu-b-110](https://gwas.mrcieu.ac.uk/datasets/ieu-b-110/) | UKB | European/440546 | 32203549 | Richardson, Tom/2020 |
| C-reactive protein  [prot-c-4337_49_2](https://gwas.mrcieu.ac.uk/datasets/prot-c-4337_49_2/) | KORA F4 study, QMDiab | European/28240269 | 501428 | 2019/Suhre K |
| Monokine Induced by Gamma Interferon | YFS and FINRISK 1997 and 2002 | European/33491305 |  | 2020/Vanessa Tan |

UKB, UK BioBank. KORA，Cooperative Health Research in the Region of Augsburg. QMDiab, the Qatar Metabolomics Study on Diabetes. Yfs, Young Finns Study. FINRISK, Finland’s National FINRISK Study.

#，T1D study data was collected from publicly available repositories, with exception of the CHOPMcGill CaCo and CHOPTDT studies (provided by C.P.1 ). Publicly available genotype data was obtained from the following sources: dbGaP for cohorts GoKinD(https://www.ncbi.nlm.nih.gov/projects/gap/cgi-bin/study. cgi?study_id=phs000088.v1.p1), EDICDCCT (https://www.ncbi.nlm.nih.gov/projects/gap/cgi-bin/study.cgi? study_id=phs000086.v2.p1),and T1DGC (http://www.ncbi.nlm.nih.gov /projects/gap/cgi-bin/study.cgi? study_id=phs000180.v1.p1); WTCCC for cohorts Type 1 Diabetes, Hypertension, Type 2 Diabetes, and Bipolar Disorder, 1958 Birth Cohort, and UK Blood Service2 ; the TwinsUK Resource for the TwinsUK cohort (http://www.twinsuk.ac.uk/data-access/).

**Finnish database disease inclusion criteria.**

Type 1 diabetes with complications

Hospital Discharge: ICD-10 E10.6, E10.6+M14.2, E10.6+M14.6, E10.7, E10.8

Hospital discharge: ICD-9 2507B|2508B

Cause of death: ICD-10 E10.6, E10.6+M14.2, E10.6+M14.6, E10.7, E10.8

Cause of death: ICD-9 2507B|2508B

KELA reimbursements: KELA codes ANY

KELA reimbursements: ICD-10 E10

Type 1 diabetes with renal complications

Hospital Discharge: ICD-10 E10.2+, E10.2+N08.39

Hospital discharge: ICD-9 2503B

Cause of death: ICD-10 E10.2+, E10.2+N08.39

Cause of death: ICD-9 2503B

Type 1 diabetes with ketoacidosis

Hospital Discharge: ICD-10 E10.1

Hospital discharge: ICD-9 2501B

Cause of death: ICD-10 E10.1

Cause of death: ICD-9 2501B

Type 1 diabetes with coma

Hospital Discharge: ICD-10 E10.00, E10.01, E10.09

Hospital discharge: ICD-9 2502B

Cause of death: ICD-10 E10.00, E10.01, E10.09

Cause of death: ICD-9 2502B

Type 1 diabetes with neurological complications

Hospital Discharge: ICD-10 E10.4+, E10.4+G59.0, E10.4+G63.2, E10.4+G73.0, E10.4+G99.0

Hospital discharge: ICD-9 2505B

Cause of death: ICD-10 E10.4+, E10.4+G59.0, E10.4+G63.2, E10.4+G73.0, E10.4+G99.0

Cause of death: ICD-9 2505B

Type 1 diabetes with peripheral circulatory complications

Hospital Discharge: ICD-10 E10.5

Hospital discharge: ICD-9 2506B

Hospital discharge: ICD-8 $!$

Cause of death: ICD-10 E10.5

Cause of death: ICD-9 2506B

Cause of death: ICD-8 $!$

Type 1 diabetes with ophthalmic complications

Hospital Discharge: ICD-10 E10.3+, E10.3+H28.0, E10.3+H36.09

Hospital discharge: ICD-9 2504B

Cause of death: ICD-10 E10.3+, E10.3+H28.0, E10.3+H36.09

Cause of death: ICD-9 2504B

Anaemias

A reduction in the number of circulating erythrocytes or in the quantity of hemoglobin.

D3_NUTRIANAEMIA

D3_HAEMOLYTICANAEMIA

D3_APLASTICANDOTHANAEMIA

Glomerular disorders

Hospital Discharge: ICD-10 N08*

Cause of death: ICD-10 N08*

Non-ischemic cardiomyopathy

[I9_HEARTFAIL_ALLCAUSE](https://r9.risteys.finngen.fi/endpoints/I9_HEARTFAIL_ALLCAUSE) and not I9_IHD
