# Supplementary figures and images for: Type 1 diabetes, its complications, and non-ischemic cardiomyopathy: a mendelian randomization study of European ancestry
Source: Cardiovasc Diabetol. 2024 Jan 13;23:31. doi: 10.1186/s12933-023-02117-7 (PMC10787423; doi:10.1186/s12933-023-02117-7)

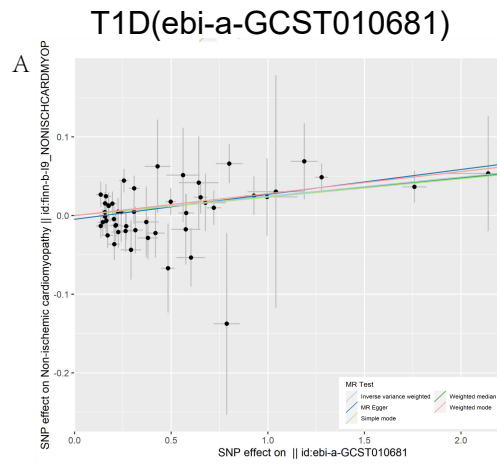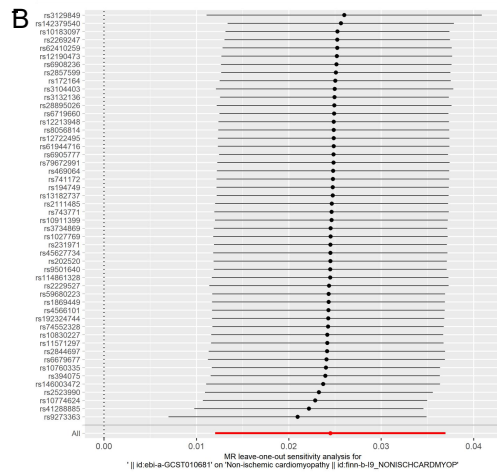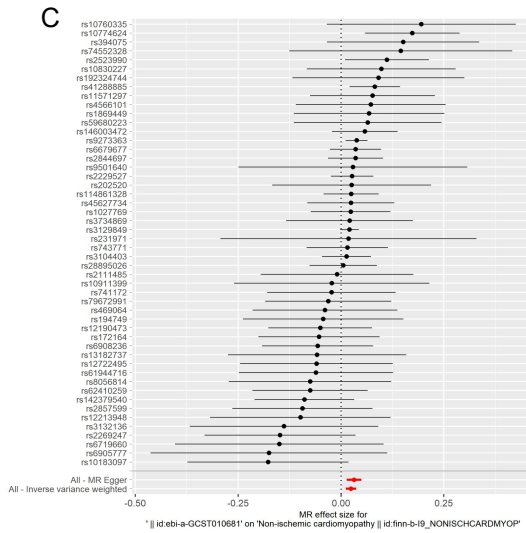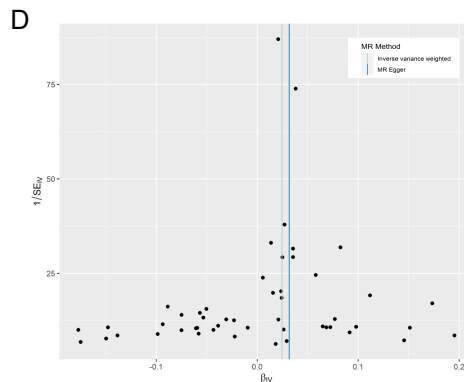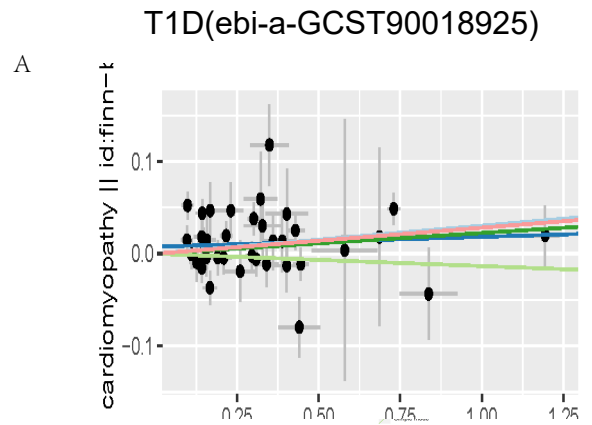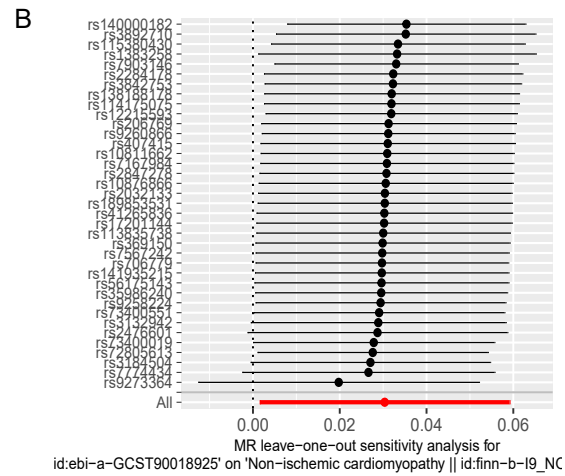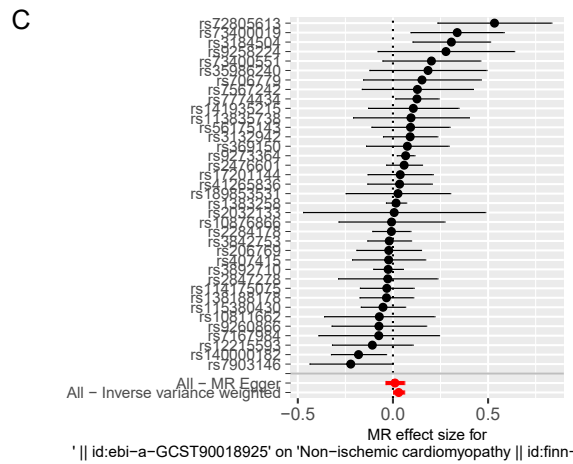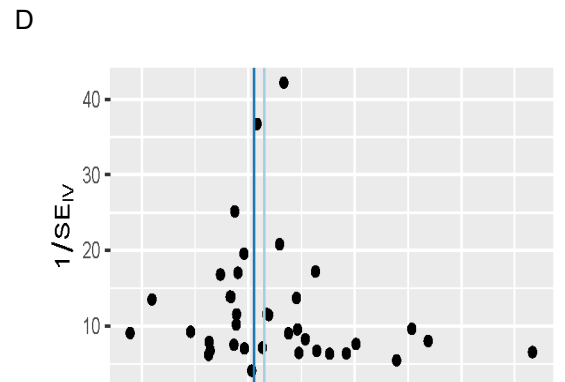

With complications

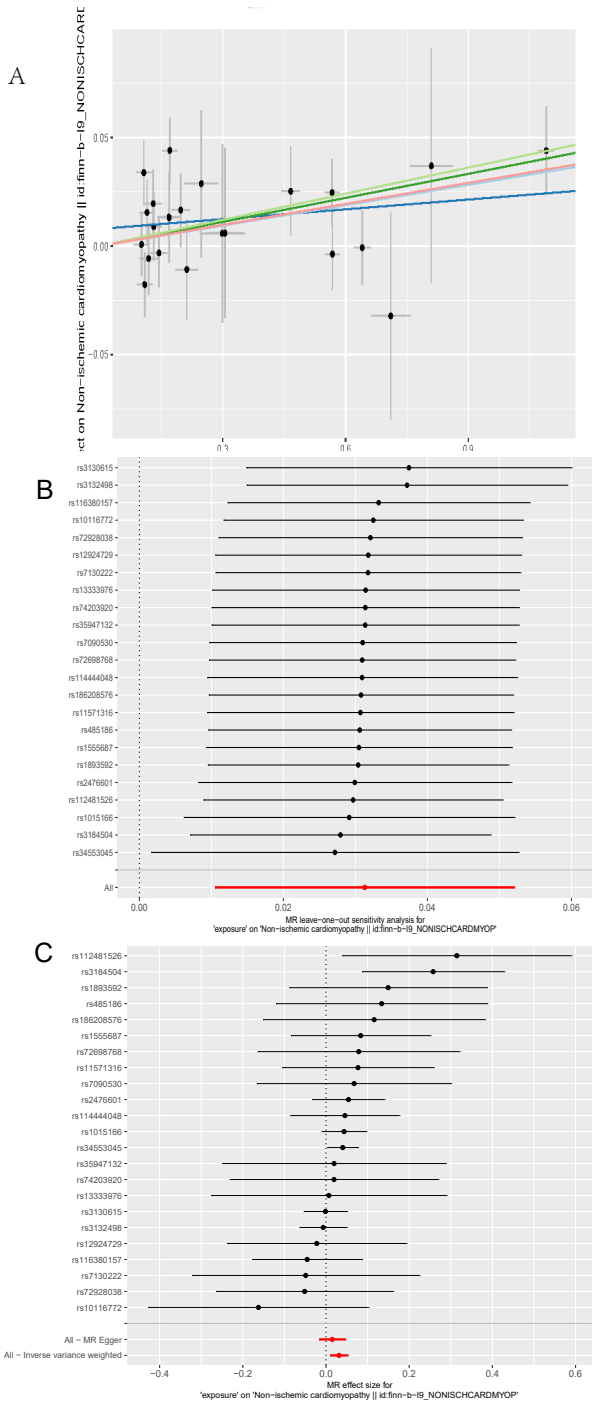

Without complications

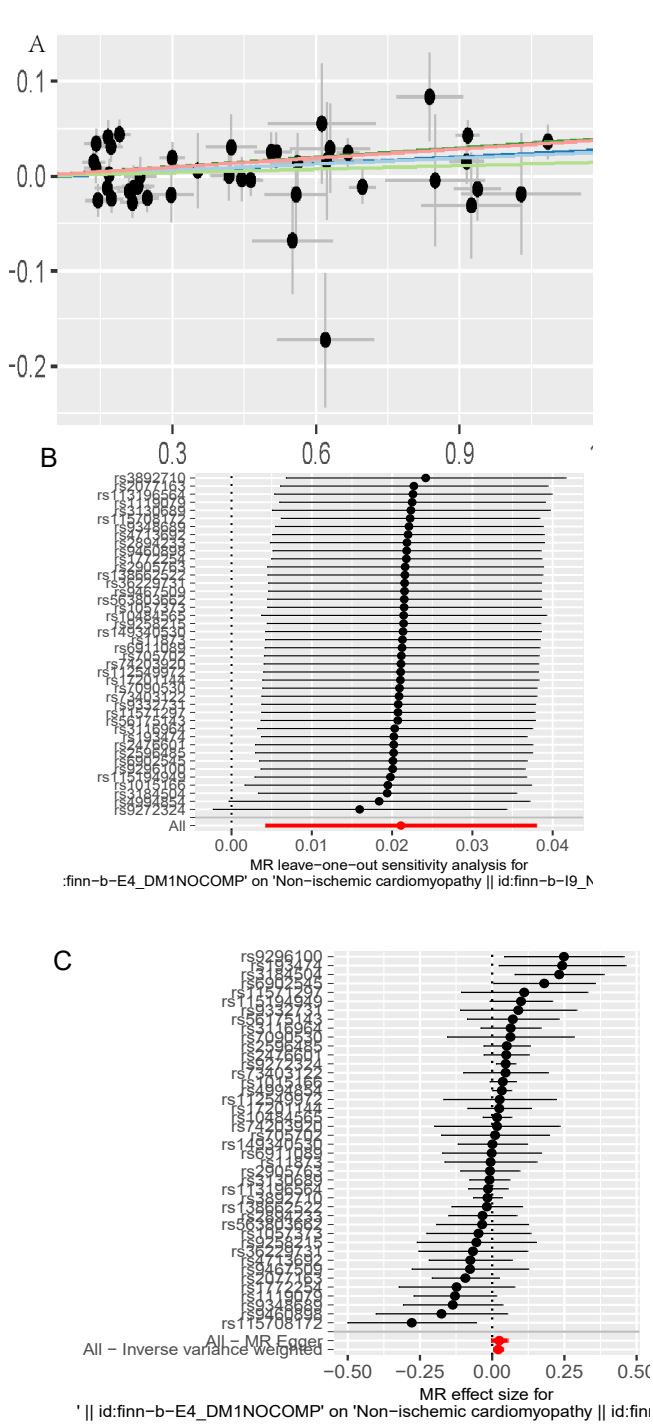

Supplement: Supplementary file 4 — Supplementary Material 4:Supplemental Figure 1. Visualization of Mendelian Randomization for T1D and T1D with Complications: A) Pleiotropy Analysis; B) Stability Analysis Utilizing the Leave-One-Out Method; C) Forest Plot Showing MR Effect Sizes Using MR-Egger and IVW; D) Funnel Plot [file 12933_2023_2117_MOESM4_ESM.pdf]
